# ASSOCIATION BETWEEN AEROBIC CAPACITY AND PERCEIVED FATIGABILITY: THE STUDY OF MUSCLE, MOBILITY, AND AGING (SOMMA)

**DOI:** 10.1093/geroni/igac059.1456

**Published:** 2022-12-20

**Authors:** Reagan Moffit, Daniel Forman, Paul Coen, Barbara Nicklas, Kyle Moored, Yujia (Susanna) Qiao, Peggy Cawthon, Nancy W Glynn

**Affiliations:** University of Pittsburgh, Pittsburgh, Pennsylvania, United States; University of Pittsburgh, Pittsburgh, Pennsylvania, United States; AdventHealth, Orlando, Florida, United States; Wake Forest School of Medicine, Winston-Salem, North Carolina, United States; Johns Hopkins Bloomberg School of Public Health, Baltimore, Maryland, United States; University of Pittsburgh, Schoold of Public Health, Pittsburgh, Pennsylvania, United States; California Pacific Medical Center Research Institute, San Francisco, California, United States; School of Public Health, University of Pittsburgh, Pittsburgh, Pennsylvania, United States

## Abstract

Peak aerobic capacity declines with age concomitant with greater fatigability and slower gait speed. We explored these relationships with cross-sectional SOMMA data (N=422, age=76.7±5.1, 57.4% women, gait speed= 0.97±0.18 m/s from a 4m walk). Participants completed a treadmill peak oxygen consumption test (VO2peak) and the self-administered Pittsburgh Fatigability Scale (PFS, range: 0-50; higher=greater fatigability). Mean VO2peak was 19.4±4.2 ml/kg/min, PFS Physical score was 16.9±8.5 points, and PFS Mental score was 8.1±8.2 points. Pearson correlations between VO2peak and PFS Physical and Mental scores were r=-0.36 and r=-0.23, respectively. For every one standard deviation higher VO2peak, PFS Physical and Mental scores were lower by 2.07 points (CI: -3.00, -1.13) and 1.10 points (CI: -2.07, -0.17), respectively, when adjusted for clinic site, age, race, sex, and self-reported physical activity. Future SOMMA analyses will evaluate a likely bidirectional association between VO2peak and fatigability, as well as examine their potential mediating effects on physical function.

